# Inhibition of lentivirus replication by aqueous extracts of *Prunella vulgaris*

**DOI:** 10.1186/1743-422X-6-8

**Published:** 2009-01-20

**Authors:** Melinda A Brindley, Mark P Widrlechner, Joe-Ann McCoy, Patricia Murphy, Cathy Hauck, Ludmila Rizshsky, Basil Nikolau, Wendy Maury

**Affiliations:** 1 Dept. Microbiology, University of Iowa, Iowa City, IA 52242, USA; 2 US Department of Agriculture-Agricultural Research Service, North Central Regional Plant Introduction Station (MPW), Ames, IA 50011, USA; 3 Department of Food Science and Human Nutrition, Iowa State University, Ames, IA 50011, USA; 4 Department of Biochemistry, Biophysics and Molecular Biology, Iowa State University, Ames, IA 50011, USA; 5 Bent Creek Institute/NCSU, The North Carolina Arboretum, 100 Frederick Law Olmsted Way, Asheville, NC 28806-9315, USA

## Abstract

**Background:**

Various members of the mint family have been used historically in Chinese and Native American medicine. Many of these same family members, including *Prunella vulgaris*, have been reported to have anti-viral activities. To further characterize the anti-lentiviral activities of *P. vulgaris*, water and ethanol extractions were tested for their ability to inhibit equine infectious anemia virus (EIAV) replication.

**Results:**

Aqueous extracts contained more anti-viral activity than did ethanol extracts, displaying potent anti-lentiviral activity against virus in cell lines as well as in primary cell cultures with little to no cellular cytotoxicity. Time-of-addition studies demonstrated that the extracts were effective when added during the first four h of the viral life cycle, suggesting that the botanical constituents were targeting the virion itself or early entry events. Further analysis revealed that the extracts did not destroy EIAV virion integrity, but prevented viral particles from binding to the surface of permissive cells. Modest levels of anti-EIAV activity were also detected when the cells were treated with the extracts prior to infection, indicating that anti-EIAV botanical constituents could interact with both viral particles and permissive cells to interfere with infectivity. Size fractionation of the extract demonstrated that eight of the nine fractions generated from aqueous extracts displayed anti-viral activity. Separation of ethanol soluble and insoluble compounds in the eight active fractions revealed that ethanol-soluble constituents were responsible for the anti-viral activity in one fraction whereas ethanol-insoluble constituents were important for the anti-viral activity in two of the other fractions. In three of the five fractions that lost activity upon sub-fractionation, anti-viral activity was restored upon reconstitution of the fractions, indicating that synergistic anti-viral activity is present in several of the fractions.

**Conclusion:**

Our findings indicate that multiple *Prunella *constituents have profound anti-viral activity against EIAV, providing additional evidence of the broad anti-viral abilities of these extracts. The ability of the aqueous extracts to prevent entry of viral particles into permissive cells suggests that these extracts may function as promising microbicides against lentiviruses.

## Background

*P. vulgaris*, commonly known as "self-heal", is a low-growing perennial herb with worldwide distribution. The herb is a member of the mint family Lamiaceae. Salves, teas, and extracts made from the plant have been used to treat wounds, inflammation, and other minor body disorders by both the Chinese and Native Americans [1,2].

Various bioactive constituents have been identified in extracts of *P. vulgaris*. These include phenolic constituents, complex carbohydrates and more hydrophobic metabolites such as triterpenes. The abundant polysaccharides present in *P. vulgaris *are readily extracted by water and have a number of reported biological activities [3,4], and several of the triterpenes have been identified with significant anti-inflammatory activity [5]. Large quantities of anti-oxidants are known to be present in aqueous *Prunella *extracts with the polyphenolic compound, rosmarinic acid, being one of the most abundant of these constituents [6,7]. Rosmarinic acid has also been shown to have anti-inflammatory activity as a result of specific inhibition of T cell signaling and an impact on glucose metabolism [8-10].

*Prunella *extracts have been reported to contain anti-viral and anti-bacterial properties, although constituents responsible for these activities are incompletely characterized to date [7,11,12]. Recent research has confirmed that anionic polysaccharides in aqueous extracts of *P. vulgaris *can decrease the replication of herpes simplex virus-1 and -2 (HSV-1, HSV-2) by preventing viral binding to cells [11,13-15]. *P. vulgaris *extracts have also been shown to contain anti-HIV activity. Studies have identified inhibition of HIV infection at steps of virus binding [16], fusion [17], reverse transcription [12], integration [18], and protease function [19]. Many of these studies identified *Prunella *antiviral activity through high through-put screens for specific viral protein targets in *in vitro *assays. While constituents in *Prunella *may be effective against these numerous anti-HIV targets *in vitro*, inhibition of the specific targets responsible for anti-HIV activity of *Prunella *in cells remains unclear. Identification of constituents of *P. vulgaris *that confer the inhibition to HIV-1 is limited to the water soluble, 10 kDa polysaccharide, Prunellin, that interferes with HIV-1 virion binding to permissive cells [16,20]. Rosmarinic acid extracted from other botanicals has proved effective against HIV-1 integrase [21], but the role of this polyphenol in the anti-retroviral activities of *Prunella *extracts has not been explored. Additional members of the Lamiaceae, such as peppermint and lemon balm, are also known to have anti-viral activities, but specific constituents responsible for those activities remain unidentified [13,22].

In this study we sought to examine the breadth of the anti-lentiviral activity of water and ethanol extracts from several *P. vulgaris *accessions by investigating their ability to inhibit replication of equine infectious anemia virus (EIAV). Water extracts of two of the accessions that had the greatest anti-viral activity were determined to interfere with virus binding and uptake. Our studies identified several different constituents present in the aqueous extracts that had significant activity against EIAV. Our findings suggest that this extract may serve as an effective microbicide against lentiviruses.

## Methods

### Growth and collection of *P. vulgaris *accessions

All *Prunella vulgaris *plant samples were provided by the North Central Regional Plant Introduction Station (NCRPIS, Ames, IA) of the Agricultural Research Service of the U.S Department of Agriculture. All samples utilized in experiments were produced from populations collected from North Carolina or Missouri in October, 2004 on a collection trip sponsored by the USDA/NCRPIS/ISU/NIH. Both seed and voucher specimens were collected from all original sites and specimens were keyed to species [31]. Seeds from accessions Ames 27664, 27665, 27666 and 27748 were germinated in Petri plates at 25°C, transferred to flats in a greenhouse (20–25°C) before final field transfer into individual control pollinated screened cages in Ames, IA. Upper flowering portions of 14 month old plants were harvested, dried for 1 week at 38 °C in a forced-air dryer with constant humidity and ground (RTC-R301ULTRAB) for analysis. All voucher specimens representing both original and regenerated populations are stored in the Ada Hayden Herbarium, Iowa State University (Ames, IA: ISC). Seeds representing both original and regenerated populations are stored at the USDA NCRPIS under controlled conditions (-20°C, 4°C for regenerated samples). Information about the specific provenance of all accessions used for the experiments is available via the Germplasm Resources Information Network database at .

### Extraction and fractionation of *P. vulgaris*

All glassware was heated at 200°C for 2 h to destroy endotoxin.

#### Water extraction

One hundred mL of boiling, endotoxin-free water was poured over 6 g of dried *P. vulgaris*. The plant material was steeped, with stirring, for 1 h and filtered through a G6 glass fiber circle (Fisher Scientific) in a Buchner funnel. The filtrate was centrifuged at 10,000 × g for 20 minutes to remove any additional particulates. The extract was lyophilized, weighed, and re-dissolved in DMSO.

#### Ethanol extraction

Six g of dried *P. vulgaris *was extracted with 500 mL of 95% ethanol via Soxhlet for 6 h. The extract was filtered, dried by rotary evaporation at < 40°C and then lyophilized. Extracts were resuspended in DMSO.

#### Size-exclusion fractionation

Two g of dry *Prunella *water extract, dissolved in 10 mL endotoxin-free water, was loaded onto a 2.5 × 75 cm Sephacryl 100HR column. Endotoxin-free water was used to elute the size-exclusion column. Two L of eluent was collected in 10 mL fractions collected for 72 h. Absorbance at 210 nm was measured for all fractions to monitor separation efficiency and identify peaks. Nine peaks were detected. Fractions composing these peaks were pooled and concentrated by lyophylization. Fractions were resuspended in endotoxin-free water.

### Endotoxin levels of extracts and fractions

All extracts and fractions were evaluated for endotoxin using the Chromogenic Limulus Amebocyte Lysate Test kit per manufacturer's instructions (Cambrex Bioscience Inc.). This assay is able to detect concentrations of endotoxin of 0.007 EU/mL or greater. All extracts had <0.007 EU/mL at the highest concentrations used in these studies. The fractions had slightly higher endotoxin levels; the highest amount of endotoxin present in the fractions when diluted for these studies was 0.023 EU/mL.

### Separation of ethanol soluble and insoluble constituents in the size fractionated fractions

Sufficient 100% ethanol was added to each fraction to yield a 95% ethanol solution. These fractions were placed in a rotary shaker at room temperature for 1h. Fractions were centrifuged at 10,000 × g for 20 min. The ethanol-soluble supernatant was decanted, and the ethanol-insoluble pellet was redissolved in endotoxin-free water. Each sub-fraction was lyophilized and weighed and resuspended in endotoxin-free water.

#### Cells and viral strains

Equine dermis cells (ED cells) (ATCC CCL57) were maintained in high glucose DMEM with 15% fetal calf serum (FCS). Primary equine umbilical vein endothelial cells (eUVEC) were also used in the EIAV studies and were maintained in high glucose DMEM with 40% FCS. All media were supplemented with penicillin and streptomycin.

Stocks of EIAV were generated in ED cells. Viral stocks of EIAV_WSU5 _[23], EIAV_MA-1 _[24], EIAV_vMA-1c _[25], EIAV_SP19 _[26], and EIAV_Th1 _[24] from ED cell supernatants were harvested from cells that were >95% positive for EIAV antigen as determined by EIAV antigen immunostaining. Supernatants were centrifuged for 5 min at 13,500 × g to remove cell debris, aliquoted, and frozen at -80°C until needed. Viral titers were determined by infection of ED cells using the single round of infection assay described below.

### Viral infection and time-of-addition studies

#### Inhibition of infectivity studies

All extracts were resuspended in DMSO, and fractions and sub-fractions were resuspended in water. 250 infectious particles of EIAV were combined with the concentrations of extracts, fractions or sub-fractions as noted in the figures. The amount of DMSO was adjusted so that equivalent concentrations of DMSO were present in all wells within an experiment. No more than 1.5% DMSO was used, as ED cell cytotoxicity was observed at higher DMSO concentrations. In experiments where extracts were studied, the quantity of DMSO was carefully controlled. As the total concentration of botanical constituents varied slightly between the different accession extracts, the quantity of constituents assayed was slightly different for each accession. The constituent concentrations that were used are noted in Table 1 and in the relevant figure legends. The extract and virus mixture were added to 5 × 10^4 ^cells/well of ED cells or eUVEC in a 48-well format resulting in a multiplicity of infection (MOI) of ~0.005. The cells were maintained for 40 h. Cells were fixed with 75% acetone/25% water fixation and immunostaining of the cells for EIAV antigens was performed as previously described [27]. The EIAV antigen-positive cells within the infected cell monolayer were counted and titers determined. IC_50 _and IC_90 _concentrations were determined using TableCurve software (Systat Academic).

**Table 1:** Concentrations of *Prunella *stocks

**Botanical**	**Concentration** **(mg/mL)**
*Extracts*:*	33.0
Ames 27664 – water	21.1
Ames 27665 – water	29.8
Ames 27666 – water	31.2
Ames 27748 – water	33.4
Ames 27664 – ethanol	34.6
Ames 27665 – ethanol	32.1
Ames 27666 – ethanol	33.7
Ames 27748 – ethanol	
*Fractions:*	100
Fraction 1	100
Fraction 2	100
Fraction 3	100
Fraction 4	100
Fraction 5	100
Fraction 6	100
Fraction 7	100
Fraction 8	100
Fraction 9	100
*Sub-fractions:*	
Ethanol soluble 1	89.1
Ethanol soluble 2	40.6
Ethanol soluble 3	53.0
Ethanol soluble 4	41.0
Ethanol soluble 5	29.1
Ethanol soluble 6	93.9
Ethanol soluble 7	63.5
Ethanol soluble 8	19.7
Ethanol soluble 9	70.5
Ethanol insoluble 1	10.9
Ethanol insoluble 2	59.4
Ethanol insoluble 3	46.9
Ethanol insoluble 4	59.0
Ethanol insoluble 5	70.9
Ethanol insoluble 6	6.2
Ethanol insoluble 7	36.5
Ethanol insoluble 8	80.3
Ethanol insoluble 9	29.6

* = all solvent used in the extraction procedure was removed from the extracts and extract material was resuspended in DMSO. All fractions and subfractions were resuspended in sterile water.

#### Inhibition of entry studies

EIAV_WSU5 _was added to ED cells at an MOI of 0.005 in ED media. DMSO or extracts of *P. vulgaris *Ames 27664 or Ames 27748 extract was added to the well at 0, 1, 2, 3, 4, 6, and 8 h following infection at a final concentration of 0.2% DMSO (66 μg/mL of Ames 27664 or 62.4 μg/mL of Ames 27748). Forty h following infection the cells were fixed, immunostained for EIAV antigen and the EIAV positive cells enumerated.

#### Cell bound EIAV studies

EIAV_WSU5 _was bound to ED cells at 4°C for 1 h to permit binding, but prevent virion internalization. The cells were warmed to 37°C and DMSO or *Prunella *extract was added to the well at 0, 1, 2, 3, 4, 6, and 8 h following temperature shift at a final concentration of 0.2% DMSO (66 μg/mL of Ames 27664 or 62.4 μg/mL of Ames 27748). Forty h following infection the cells were fixed, immunostained for EIAV antigens and EIAV-positive cells enumerated.

#### Internalization studies

EIAV_WSU5 _was bound to ED cells at 4°C for 1 h to permit binding, but prevent virion internalization. Unbound virus was removed, new media replaced, and the cells shifted to 37°C to promote internalization. At 0, 1, 2, 4, and 6 h following temperature shift, the cells were washed briefly in citrate acid buffer (pH 3.0) to inactivate any non-internalized virions. The citrate buffer was removed and cells were washed twice, and medium contain 0.2% of DMSO, 66 μg/mL of Ames 27664 extract, or 62.4 μg/mL of Ames 27748 extract was added to determine if the extracts had any inhibitory effect on virions that had already been internalized.

#### Virion stability studies

EIAV viral stock was incubated in DMEM with 10% fetal calf or DMEM 10% fetal calf plus 132 μg/mL of Ames 27664 extract or 126 μg/mL of Ames 27748 extract. The virus stock was maintained at 37°C and used to infect ED cells at various time points following extract exposure. The final concentration of *Prunella *when diluted on the cells was 0.44 μg/mL of Ames 27664 extract or 0.42 μg/mL of Ames 27748 extract. At 40 h following initiation of infection, the cells were fixed and immunostained for the production of EIAV proteins.

#### Viral binding assay

Virus was mixed with 132 μg/mL of Ames 27664 extract or 126 μg/mL of Ames 27748 extracts (final concentration of 0.4% DMSO) or fractions (100 ug/mL) and incubated with ED cells (MOI of 2) at 4°C for 2 h to permit binding, but prevent virion internalization. Unbound virions were removed and cells were washed with phosphate buffered saline (PBS) three times to ensure all unbound virions were removed from the cells. Each well was lysed in 50 μL of lysis buffer (50 mM Tris HCl (pH 8), 120 mM NaCl, and 0.5% NP40, and 1 U/mL of protease inhibitor cocktail (Sigma). The lysates were analyzed by immunoblotting for the presence of viral capsid to indicate virus binding as described below. Blots were re-probed for cellular β-tubulin to normalize for cellular input.

#### Inhibition of virion infectivity studies

10^5 ^infectious particles of EIAV_wsu5 _were incubated at room temperature for 10 min with *P. vulgaris *Ames accession 27664 aqueous extract. Following the incubation, dilutions of the incubated virus were added to ED cells in a 48-well format and appropriate concentrations of extract were maintained on the cells for the duration of the experiment. Cells were fixed at 40 h following infection and immunostained as described above. Wells with serial dilutions containing between 10 and 250 virus positive cells were enumerated and back-calculations were made to obtain the numbers of infectious units of virus/mL.

#### Immunoblotting

Cell lysates were run on NuPAGE Novex Bis-Tris Mini Gels (Invitrogen) and transferred to nitrocellulose. EIAV capsid was detected using the 2085 sera (1:10000) and secondary anti-horse antisera (1:10000) that was used for immunostaining. Tubulin was detected by the E7 monoclonal antibody (1:2000) (NIH Developmental Studies Hybridoma Bank, University of Iowa) and sheep anti-mouse HRP secondary (GE Healthcare) (1:50,000). All immunoblots were visualized using WestDura (Pierce).

#### Sucrose-gradient centrifugation

Sucrose step gradients were prepared by layering 250 μL aliquots of decreasing concentrations of sucrose (20%–60%) into 3 mL ultra centrifugation tubes. The gradients were allowed to equilibrate at 4°C for at least 3 h. Virions were treated with extracts (0.4%), 0.5% Triton-X 100 or DMSO for 1 h at 37°C and loaded onto the top of the gradients. Tubes were centrifuged for 16 h at 40,000 rpm in a SW60 rotor at 4°C and stopped without a brake. Two hundred and fifty microliter aliquots were collected beginning from the top of the tube and stored at -80°C until analyzed by immunoblotting.

#### Cell viability studies

Cells were plated and treated with extracts as described above. Forty h following treatment cell viability was monitored by ATPLite Assay (Packard Biosciences) per manufacturer's instructions.

#### Statistical analysis

Studies were performed at least three independent times except where noted in the figure legends. Means and standard errors of the mean are shown. Student's t-test was used to evaluate the statistical differences between treatments, utilizing the two-tailed distribution and two-sample equal-variance conditions. P-values were assessed by comparing the level of infectivity with treatment to the level of cytoxicity seen with that same treatment. P-values for viability were assessed by comparing the level of viability with treatment to the level of viability seen with DMSO or control. A significant difference was determined by a p-value of < 0.05 and significance was identified in each figure. If the p-value was > 0.05, the data were not considered statistically significantly different.

## Results

### Aqueous extracts from *P. vulgaris *inhibit EIAV infectivity without significant cell toxicity

**Figure 1:**
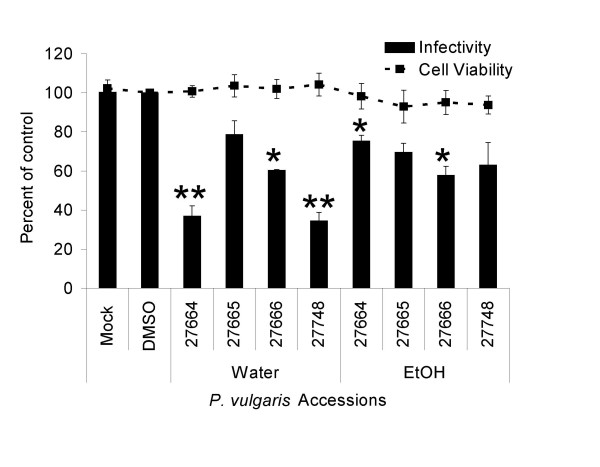
**Water extracts of *P. vulgaris *inhibit lentiviral infectivity with low cell toxicity**. DMSO, water extracts and ethanol extracts of *P. vulgaris *were diluted in media to 0.2% (water extracts: 66 μg/mL of Ames 27664, 42.2 μg/mL of Ames 27665, 59.6 μg/mL of Ames 27666, or 62.4 μg/mL of Ames 27748 and ethanol extracts: 66.8 μg/mL of Ames 27664, 69.2 μg/mL of Ames 27665, 64.2 μg/mL of Ames 27666, or 67.4 μg/mL of Ames 27748). Equivalent quantities of EIAV_WSU5 _were added to each well of ED cells along with the diluted extracts. Forty h following infection, cells were fixed and immunostained for viral antigen. Cell-viability studies were performed in parallel. Cell viability and virus infectivity are shown as a ratio of the values in the presence of the extracts divided by the DMSO control. Shown are the averages and standard errors of three experiments performed in triplicate. *, p < 0.05; **, p < 0.001.

Water and ethanol extractions were prepared from four accessions of *Prunella vulgaris*. Three of the accessions (Ames 27664, 27665 and 27666) were collected in western North Carolina; Ames 27748 was collected in Missouri. Ames 27664 and 27748 were obtained from disturbed roadside areas whereas the other two accessions were collected from more remote, forested habitats. These extracts were screened for their ability to inhibit EIAV_WSU5 _in a single-round infection assay. Extracts and virus were diluted in media and immediately incubated with cells. Forty h following infections, the cells were fixed, immunostained for expression of EIAV antigens and antigen-positive cells enumerated to determine the level of viral infection (Fig. 1). Although both ethanol and water extracts demonstrated some ability to inhibit EIAV_WSU5_, water extracts contained the largest quantities of anti-viral activity. At the concentrations tested, all extracts had little or no cytotoxicity. Aqueous extracts of Ames 27664 and 27748 had the most significant anti-viral effect against EIAV. While the aqueous extracts of Ames 27664 and 27748 contained slightly greater concentrations of constituents than the other two aqueous extracts, these modestly higher concentrations could not solely explain the better activity of these accessions since serial dilutions of extracts from Ames 27664 and 27748 still had more anti-viral activity against EIAV than undiluted aqueous extracts from Ames 27665 and 27666 (Additional file 1). Thus, aqueous extracts of Ames 27664 and 27748 were further studied to determine the block in EIAV replication.

**Figure 2:**
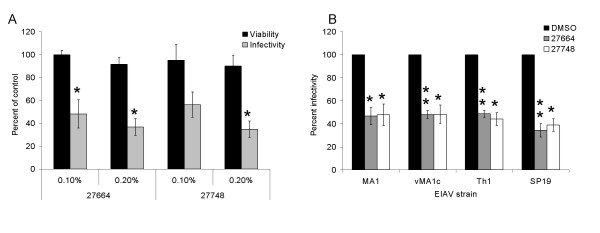
**EIAV inhibition by water extracts of *Prunella *Ames 27664 and 27748 is not cell type or strain specific**. A) EIAV infections of UVECs. DMSO and water extracts of Ames 27664 and 27748 were diluted in media to 0.1% (33 μg/mL of Ames 27664 or 31.2 μg/mL of Ames 27748) or 0.2% (66 μg/mL of 27664 or 62.4 μg/mL of 27748). EIAV_WSU5 _virus was added to the diluted extracts and immediately used to infect eUVECs. B) Inhibition of infection of four EIAV strains was evaluated in ED cells in the presence of 0.2% *P. vulgaris *aqueous extracts of Ames 27664 and 27748 or DMSO. Forty h following infection, cells were immunostained for EIAV antigen. Parallel cultures that were treated with extract, but not infected were evaluated for cell viability. Shown are the ratios of the values in the presence of the extracts divided by the DMSO control. Shown are the averages and standard errors of three experiments performed in triplicate. *, p < 0.05; **, p < 0.001.

To ensure that the effects seen in the initial study were not viral-strain or cell-type specific, water extracts of *P. vulgaris *Ames 27664 and 27748 were tested for inhibition of EIAV replication in primary cells. Primary equine umbilical vein endothelial (eUVEC) cells were infected with EIAV_WSU5 _in the presence of the extracts and EIAV infection was inhibited to a similar degree as observed in ED cells (Fig. 2A). Extracts also showed no cytotoxicity in the primary cells.

Aqueous extracts of Ames 27664 and 27748 were also tested for their ability to inhibit a variety of EIAV strains (Fig. 2B). Two tissue-culture adapted strains, EIAV_MA1 _and EIAV_SP19, _as well as a field isolate, EIAV_Th1_, and a variant superinfecting strain, EIAV_vMA1c_, were effectively inhibited by the extracts. Previous studies have demonstrated that EIAV_vMA1c _enters ED cells through an alternative pathway compared to its parental strain EIAV_MA1 _[28-30]. EIAV_vMA1c _enters ED cells through plasma membrane fusion whereas EIAV_MA1 _and other wild-type strains of EIAV enter ED cells through interaction with the cellular receptor ELR1 that is mediated by a low-pH dependent, clathrin-mediated endocytosis event. The observation that the *P. vulgaris *extracts inhibit both the wild-type strains and variant strain equivalently suggest that *Prunella *anti-viral activity is broadly inhibitory and does not block specific viral-entry events, such as viral glycoprotein/ELR1 interactions.

**Figure 3:**
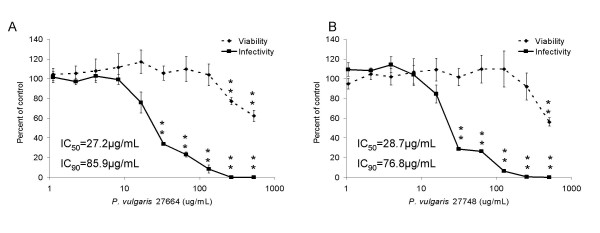
**Dose dependent inhibition of aqueous extracts of *Prunella *Ames 27664 and 27748 to inhibit EIAV infection**. Increasing concentrations of *P. vulgaris *aqueous extracts A) Ames 27664 and B) Ames 27748 were evaluated for the ability to inhibit EIAV_WSU5 _infection (solid lines). Parallel cell viability studies were performed (dotted lines). Shown are the ratios of the values in the presence of the extracts divided by the DMSO control. Shown are the averages and standard errors of three experiments performed in triplicate. **, p < 0.001.

To examine the inhibition of the *Prunella *aqueous extracts more closely, dose response curves were generated and the concentrations of extracts required to inhibit 50% and 90% of viral infection (IC_50 _and IC_90_, respectively) determined. Fifty percent of EIAV_WSU5 _infectivity is inhibited with 27.2 μg/mL of *Prunella *Ames 27664 and 28.7 μg/mL of *Prunella *Ames 27748, and ninety percent is inhibited by 85.9 μg/mL and 76.8 μg/mL, respectively (Fig. 3A and 3B). While the dose of extract needed to inhibit 50% of cell viability (LD_50_) could not be determined due to limited observed cytotoxicity, an approximate 40% reduction in viability was observed at the highest dose tested (600 μg/mL) suggesting that the therapeutic window (LC50/IC50) was more than 20-fold.

### *Prunella *extracts primarily inhibit early steps in the EIAV life-cycle

**Figure 4:**
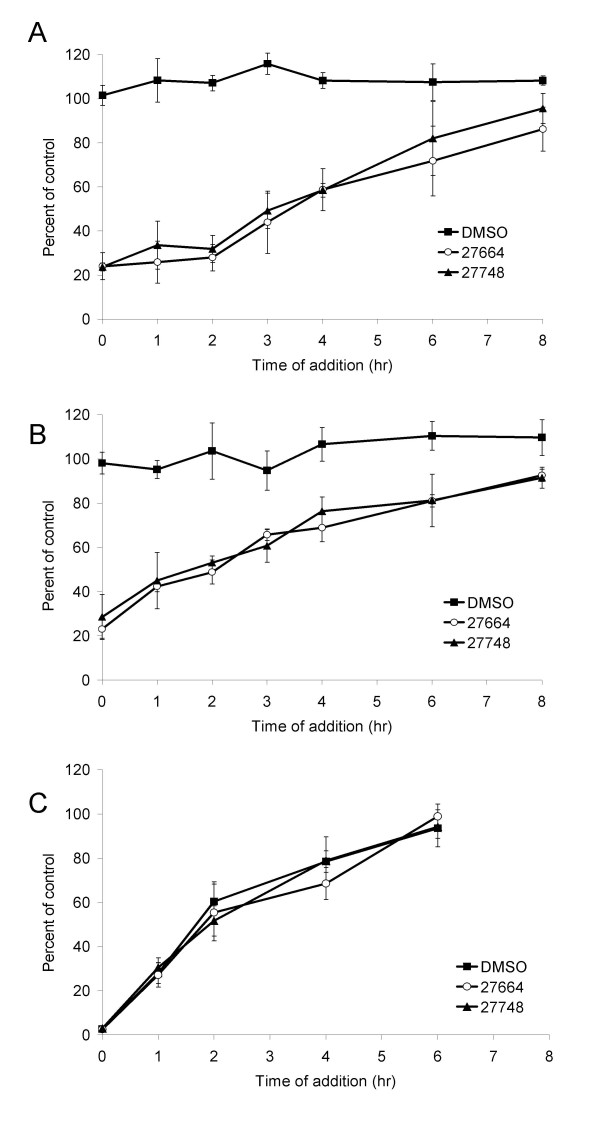
**Early steps in the EIAV life cycle are inhibited by *Prunella *aqueous extracts**. A) Time frame of extract inhibition of EIAV infection. ED cells were infected with EIAV_WSU5 _virus and, at times noted following infection, DMSO, Ames 27664 extract, or Ames 27748 extract was added to the cells to a final concentration of 0.2% (66 μg/mL of Ames 27664 or 62.4 μg/mL of Ames 27748). B) Time frame of extract inhibition of EIAV_WSU5 _that was previously bound to ED cells. Viral particles were bound to ED cells at 4°C for 1 h. Unbound virus was removed and cells were shifted to 37°C. At the times indicated, DMSO, Ames 27664 extract or Ames 27748 extract was added to the cells to a final concentration of 0.2%. The percent of infected cells was determined by dividing the number of EIAV antigen positive cells in the presence of extract compared to the number of EIAV antigen positive cells in the presence of DMSO at time zero. C) Time frame of extract inhibition of EIAV_WSU5 _following virion internalization. Virions were bound to ED cells at 4°C for 1 h, unbound virions were removed, fresh media was replaced, and cells were permitted to internalize at 37°C. At the time points indicated, the cells were washed with citric acid buffer to inactivate any non-internalized virions, washed and media containing DMSO, Ames 27664 extract, or Ames 27748 extract was added (0.2%). Data represent the average and standard error of three experiments performed in duplicate. *Prunella *extracts significantly decreased EIAV infectivity compared to DMSO control at 0, 1, 2, 3, and 4 h time points in panels A and B (p < 0.05). Differences observed at 6 and 8 h were not statistically significant.

To determine step(s) during the viral life cycle that are inhibited by the extracts, time-of-addition experiments were performed. In the first set of experiments, ED cells were infected with EIAV_WSU5 _and at six time points following infection, aqueous extracts were added to the infected cells. Addition of extracts at 1–4 h following initiation of infection effectively inhibited virus replication with a decreasing impact of extract addition over time (Fig. 4A). We have previously shown that EIAV binds to cells within six h of infection [30]. Here we demonstrate that by six h, the addition of the extracts did not have a statistically significant effect on EIAV infectivity. This finding indicated that the inhibition by *Prunella *extract was occurring prior to or during virus entry.

Because the aqueous extracts were inhibiting early steps in the viral life-cycle, we sought to determine if the extracts interfered with entry steps either prior to or following virus binding to permissive cells. Virus was pre-bound to ED cells at 4°C to permit binding, but prevent internalization. After the one h binding step, unbound virions were removed and the cells shifted to 37°C to promote internalization. Extracts were added at various times following the temperature shift to 37°C. Under these conditions, our previous studies have demonstrated that EIAV is internalized from the surface of ED cells within four h [29]. When EIAV was pre-bound, EIAV infectivity at early times of infection (1–4 h) was less sensitive to *Prunella *inhibition than when virus was not pre-bound (Fig. 4B and Table 2). For instance in the absence of a pre-binding step, the addition of *Prunella *extracts at 2 h resulted in 77% inhibition of infectivity. In contrast, if the virus was pre-bound, 53% of the virus was sensitive to *Prunella *inhibition. This finding suggested that constituents in *Prunella *aqueous extracts were interfering to some extent with virus binding to ED cells and a pre-binding step decreased the inhibition observed at early time points. However, smaller, but significant reduction in viral infectivity was also observed following a pre-binding step, indicating *Prunella *aqueous extracts interfere with post-binding events as well.

**Table 2:** Percent of virus added that is sensitive to *Prunella *extracts

	**Time of addition (hr)**						
							

**Treatment**	**0**	**1**	**2**	**3**	**4**	**6**	**8**

**No pre-binding**	78	79	77	69	50	31	17

**Pre bound virions**	72	52	53	32	34	29	18

**Internalized virions**	0	-1	7	nd	5	-3	nd

Percent of virions sensitive to the extracts was calculated by subtracting the infectivity in the presence of the extracts from the DMSO control at each time point. Data from the extracts 27666 and 27748 were averaged. nd, data not determined.

We also tested the ability of *Prunella *aqueous extracts to inhibit virions that have been internalized from the cell surface. Virions were bound to ED cells at 4°C, unbound virions were removed, fresh media replaced and the cells shifted to 37°C to promote virion internalization. At 0, 1, 2, 4, and 6 h following 37°C temperature shift, the cells were treated with citric acid buffer that inactivates all virions remaining on the cell surface. The cells were washed and media containing DMSO or extracts added to the cells and maintained for the 40 h infection. Internalized virions were not impacted by *Prunella *extracts (Fig. 4C). In total, our data suggest that the *Prunella *extracts inhibit EIAV infectivity by interfering with virus binding and subsequent requisite steps that occur prior to virion internalization. However, once the virions are internalized, the extract was not inhibitory.

**Figure 5:**
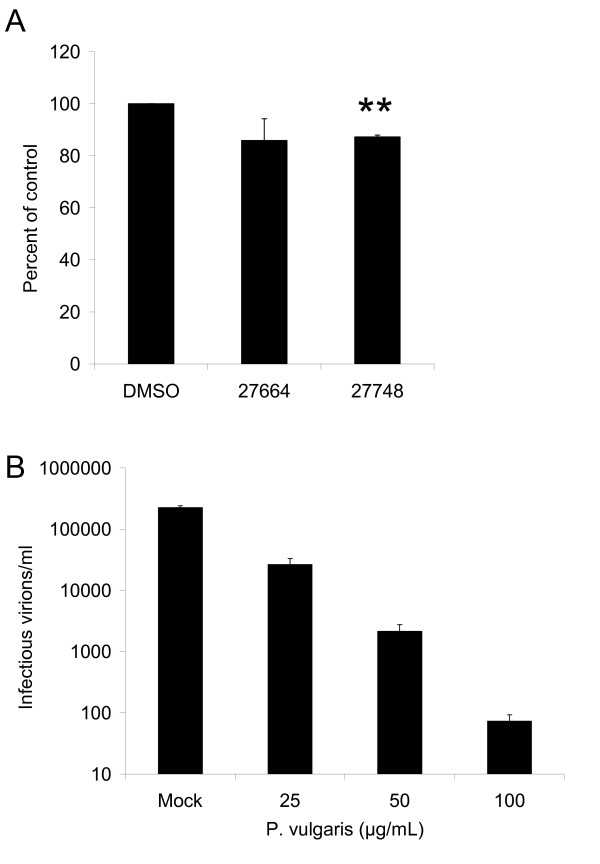
***Prunella *extracts inhibit EIAV infectivity primarily by acting on viral particles**. A) Ability of cell-associated *Prunella *extracts to inhibit EIAV infection. Extracts were incubated with ED cells and removed prior to the addition of EIAV. EIAV infection was evaluated at 40 h following infection. B) Ability of *Prunella *extracts to inhibit EIAV. 2.2 x 10^5 ^EIAV_WSU5 _particles were incubated with 25, 50 or 100 μg/mL extract from *Prunella *Ames 27664 extract for 10 minutes at room temperature. Viral stocks were diluted in media containing the appropriate concentration of extract and plated onto ED cells. Forty h following infection the cells were fixed, immunostained for EIAV antigen and enumerated. Shown are the numbers of infectious virions/ml. Data represent the average and standard error of three experiments performed in duplicate (A) or triplicate (B). Extracts significantly reduced EIAV virion infectivity at all concentrations tested.

Next we wanted to determine if exposing the cells to the extracts, without exposing the virions, could inhibit EIAV replication. ED cells were incubated with the extracts for two h. The medium was changed and cells infected with EIAV_WSU5_. Pre-exposure of cells to the extracts from *Prunella *Ames 27748 reduced the level of infectivity by 15% which was statitistically significantly different from the control (Fig. 5A). The modest inhibition observed with extracts of Ames 27664 was not found to be statistically significant because of larger amounts of experimental variation in studies performed with this extract. The limited antiviral activity found in this experiment is consistent with the time-of-addition studies, suggesting that the extracts need to come in direct contact with the virions for the most robust inhibition.

To determine the reduction in particle infectivity by *P. vulgaris *extracts, we incubated 10^5 ^infectious virions of EIAV_WSU5 _with 25 to 100 μg/mL of extract for 10 min at room temperature. Virions were serially diluted in media containing the same concentration of extract and plated on ED cells. Virus infectivity was evaluated 40 h later. Incubation of virions with aqueous extract has a profound impact on virion infectivity with 100 μg/mL of extract resulting in greater than 3000-fold reduction in infectivity, indicating that the majority of the anti-viral effect seen is caused by the extracts interacting with the viral particles directly, rather than inhibiting later steps in the viral life-cycle (Fig. 5B).

### *Prunella vulgaris *extracts inhibit virion binding to cells

**Figure 6:**
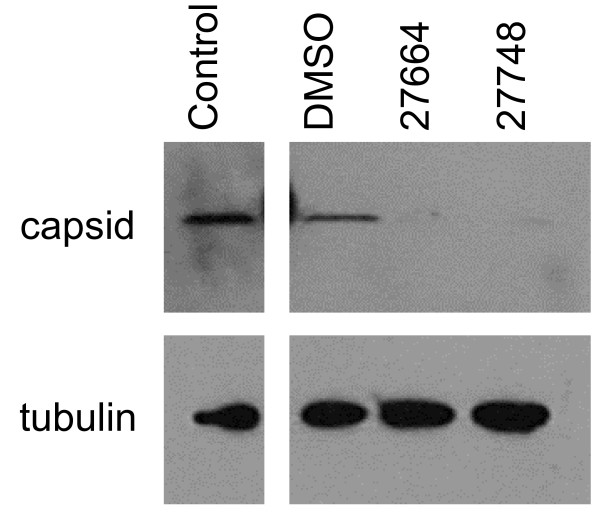
***Prunella *extracts inhibit virion binding to cells**. EIAV_WSU5 _virions mixed with the *Prunella *(132 μg/mL of Ames 27664 or 126 μg/mL of Ames 27748) extracts or DMSO and added to ED cells at 4°C to promote binding but prevent internalization. After a two h binding period, the unbound virions were removed, cells were washed and lysed. Cell lysates were immunoblotted for EIAV Gag proteins to detect bound virions, and tubulin served as a loading control. The experiment was repeated three times. A representative blot is shown.

To determine if loss of virion infectivity was the result of reduced ability of virions to bind to permissive cells in the presence of *Prunella *extract, EIAV was incubated in the presence of the extracts on cells for 2 h at 4°C. These conditions allow binding, but prevent particle internalization. Unbound virions were removed and cells and virions associated with the cells were lysed. Lysates were examined for the presence of the viral protein, Capsid, to determine if the extracts reduced EIAV binding to the permissive cell population. When incubated with the DMSO control, EIAV Capsid protein was found in the lysates (Fig. 6). When the virions were exposed to the *Prunella *extracts, little or no Capsid was found to be associated with the lysates, indicating the extracts inhibit this initial step of viral replication.

### Virions treated with *Prunella *extract are intact

**Figure 7:**
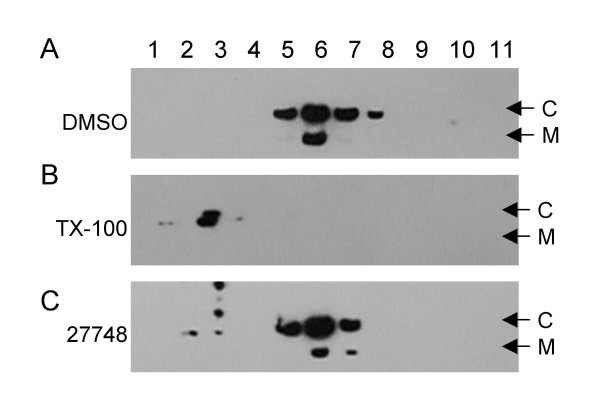
***Prunella *extracts do not destroy EIAV particles**. EIAV_WSU5 _virions were incubated with DMSO, Triton X-100 (0.5%), or aqueous extracts of *Prunella *extract (0.4% final concentration, 132 μg/mL of Ames 27664 or 126 μg/mL of Ames 27748) for 1 h at 37°C. The treated virions were density banded on a 20–60% sucrose gradient. Eleven fractions were collected and immunoblotted for EIAV Gag proteins, Capsid (C) and Matrix (M). The experiment was repeated three times and a representative blot is shown.

Because the extracts were directly inhibitory to the viral particle binding to permissive cells, we wanted to determine if the extracts destroyed the viral particle thereby rendering them non-infectious and unable to bind to permissive cells. Immunoblots of density gradient separated, extract-treated viral particles were performed to evaluate the integrity of the virions. Viral Gag proteins were found in fractions 5, 6, 7 and 8 when the virions were treated with DMSO (Fig. 7A). Triton X-100 lysis of the virions resulted in the presence of Capsid protein in the top three fractions (Fig. 7B). The vast majority of Gag proteins was also found in fractions 5, 6, and 7 after treated with the *Prunella *extracts in a manner similar to that of the DMSO control (Fig. 7C). However, Gag proteins in *Prunella *treated samples were no longer present in fraction 8 and a modest percentage of these proteins was now found in the top of the gradient. These findings suggest that in general extracts did not destroy the virions or dramatically alter their density.

### Fractionation of whole-plant extracts

**Figure 8:**
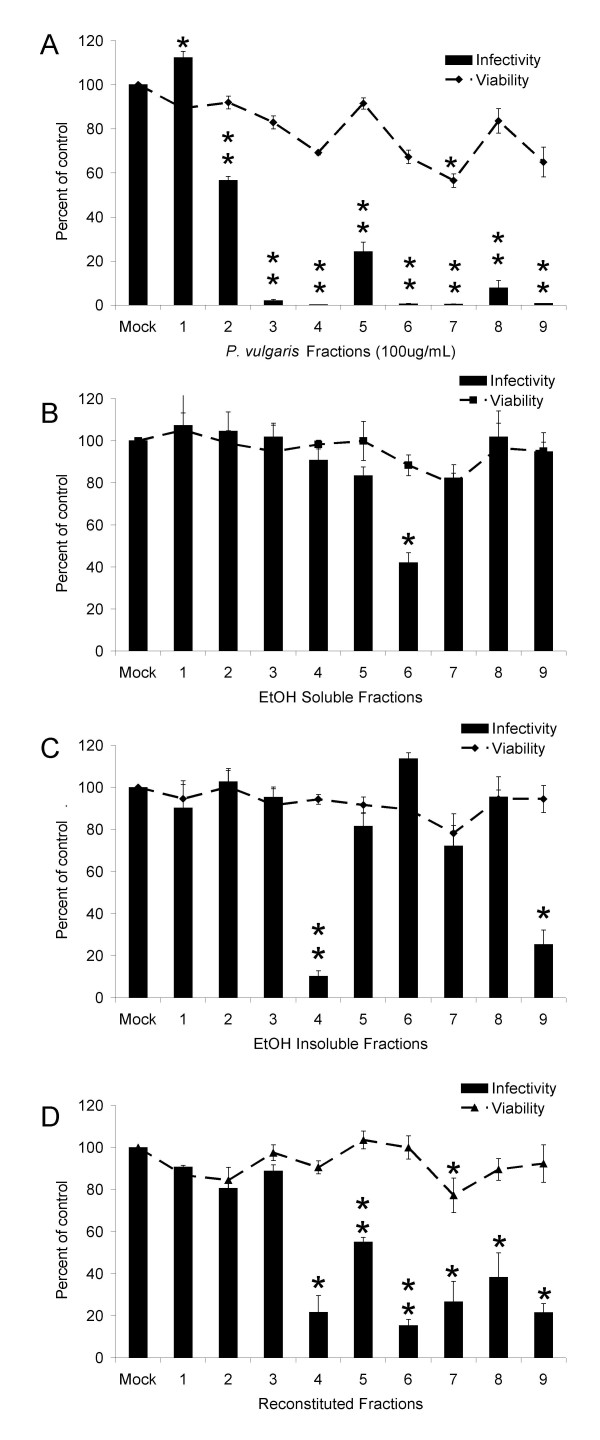
**Fractionation segregates anti-viral constituents**. ED cells were infected with EIAV_WSU5 _in the presence of the size exclusion fractions (100 μg/mL) (A), ethanol-soluble sub-fractions (B), ethanol-insoluble sub-fractions (C), or a combination of both the ethanol soluble and insoluble fractions at concentrations that reconstitute the original fractions (D). Forty h following infection, cells were fixed and immunostained for viral antigen. Cell viability studies were performed in parallel. Cell viability and virus infectivity are shown as a ratio of the values in the presence of the fractions divided by the control. Data represent the average and standard error of three experiments performed in triplicate. *, p < 0.05; **, p < 0.001.

Aqueous extracts of *Prunella *would be anticipated to contain abundant amounts of carbohydrates, phenolics and other water-soluble constituents. To begin to identify *Prunella *constituents within the aqueous extracts that are important for the anti-EIAV activity, the aqueous extract of *Prunella *Ames 27748 was separated by a Sephacryl 200 size-exclusion chromatography column into nine fractions. Both the distinct color of each of the fractions as well as LC/MS analysis of the fractions indicated that successful separation of *Prunella *constituents was achieved (data not shown). These fractions were resuspended in endotoxin-free water at a stock concentration of 100 mg/mL and were tested for anti-EIAV activity. Surprisingly, fractions 3–9 contained potent anti-viral activity and fraction 2 had some inhibitory activity (Fig. 8A). Significant levels of cytotoxicity were observed with 100 μg/mL of fraction 7. Cytotoxicity of fraction 7 was in contrast to what we had observed with similar concentrations of aqueous extract suggesting that either cytotoxic compounds were being concentrated in this fraction or that the separation of constituents resulted in greater cytotoxicity by some metabolites.

To further characterize the anti-viral constituents in the fractions, ethanol precipitation of the nine fractions was performed to separate ethanol-soluble and insoluble compounds. Constituents present in the sub-fractions were weighed and resuspended in endotoxin-free water at concentrations that represented the same ratio between the soluble and insoluble constituents present in the original fraction (see Table 1 for concentrations). A single ethanol-soluble fraction, Fraction 6, showed significant inhibition of EIAV (Fig. 8B). Ethanol-insoluble sub-fractions 4 and 9 displayed potent anti-EIAV activity (Fig. 8C). Interesting, the ethanol precipitation of fractions 2, 3, 5, 7 and 8 resulted in complete loss of anti-EIAV activity in either sub-fraction.

To determine if the ethanol precipitation destroyed the anti-viral activity or if multiple constituents that were separated during sub-fractionation were required for activity, we performed reconstitution experiments. The soluble and insoluble sub-fractions were added together at concentrations found in the original fractions and tested for anti-viral activity (Fig. 8D). The anti-viral activity seen in the original fraction 2 and 3 was lost after sub-fractionation and was not reconstituted. Reconstitution of fractions 4 and 9 did not enhance the anti-viral activity over that observed with the ethanol insoluble sub-fraction alone. Fraction 6 from the ethanol-soluble sub-fraction displayed anti-viral activity; however, after reconstitution, anti-viral activity was enhanced. Surprisingly, anti-viral activity was restored in fractions 5, 7 and 8 after reconstitution, suggesting synergy between constituents is required for the anti-viral activity of these fractions.

## Discussion

This study identified anti-viral activity against the lentivirus EIAV in aqueous extracts of *P. vulgaris*. The primary mechanism of inhibition of viral replication targeted viral entry. The extracts dramatically reduced infectivity when incubated with the virions alone and interfered with the ability of virus to bind to permissive cells. However, entry of EIAV particles that were pre-bound to ED cells prior to exposure to the extract was also inhibited, suggesting the anti-viral activity was not limited to inhibition of viral binding, but also prevented additional external events that are required for subsequent internalization and/or fusion. These extracts were not blocking specific interactions between EIAV and permissive cells such as the interaction of the gp90 glycoprotein and the cellular receptor ELR1 since the extracts effectively blocked infection of EIAV_vMA-1c _which can utilize a different cellular receptor [30].

Our fractionation studies indicated that numerous *Prunella *constituents were present in the aqueous extracts that have inhibitory activity against EIAV. To begin to identify the individual constituents responsible for the anti-viral activity, we separated the aqueous extracts by size-exclusion chromatography and subsequently separated those fractions into ethanol-soluble and insoluble components. Initial fractionation of the extract by size was not highly informative since 8 of the 9 fractions retained anti-viral activity. With five of these fractions, separation of constituents by ethanol precipitation resulted in loss of all activity; whereas, the ethanol-soluble material from one fraction had anti-viral activity and ethanol-insoluble sub-fractions from two other fractions were active. The anti-viral activity found in fractions 4 and 9 were ethanol-insoluble suggesting that carbohydrates may be responsible for the activity. The activity found in fraction 6 was ethanol-soluble and therefore is likely to be polyphenolic in nature. Of the five fractions where activity was lost upon sub-fractionation, activity was reconstituted when the ethanol-soluble and insoluble sub-fractions were combined to regenerate fractions 5, 7 and 8. Our findings demonstrated that synergy between ethanol-soluble and insoluble constituents is necessary for the anti-viral activity in these fractions. Botanical constituents responsible for these anti-viral activities remain to be identified.

The extracts were found to effectively inhibit a range of EIAV strains from field isolates to a laboratory variant that can use a different cellular receptor to enter cells. The inhibition was observed in primary cells as well as a cell line. The breadth of the anti-viral activity of *Prunella *extracts was not unexpected since *Prunella *aqueous extract had previously been characterized to inhibit both the distantly related lentivirus HIV-1 and the unrelated DNA virus HSV-1 [11,12,16,17]. One of these studies identified a 10 kDa sulfated carbohydrate Prunellin from *Prunella *extracts that inhibited HIV-1 entry [16,20]. A carbohydrate of approximately that same size was responsible for inhibiting HSV-1 entry into cells [15]. While not definitively demonstrated, it is likely that Prunellin is also responsible for the anti-HSV-1 activity. Prunellin may be responsible for anti-EIAV activity found in ethanol-insoluble fraction 4 or 9. However, the ability of ethanol-insoluble constituents present in two non-contiguous fractions to robustly inhibit infectivity implicates additional, currently unidentified carbohydrates in the anti-viral activity against EIAV.

Extracts from other Lamiaceae species have been shown to bind to HIV-1 particles interfering with HIV-1 entry into permissive cells [22]. HIV-1 virions in the presence of extracts from lemon balm were shown to be denser in a sucrose gradient than virions in absence of extracts suggesting that extracts either altered the structure of the particle resulting in enhanced particle density or the constituents were bound to virions making the particles denser [22]. In our study, EIAV virions were not destroyed by treatment with the extracts. Nor did we observe a change in EIAV virion density as had been reported for HIV-1/lemon balm extracts [22]. While, it is likely that the *Prunella *extract binds to EIAV particles reducing productive, but non-specific interactions with target cells, this interaction did not significantly alter virion density. In addition, lemon balm extracts were not effective against HIV particles that were pre-bound to cells [22], a finding distinctly different from our observations that both binding events and post-binding events were affected by *Prunella *extracts.

The aqueous extracts from two of the *Prunella *accessions were significantly more inhibitory than extracts from two other accessions that were evaluated despite the fact that all four accessions were field grown in Iowa under similar conditions. The concentrations of the extracts used in this study could not account for this observation suggesting that there is extensive genotypic variation of *Prunella *in the field. The two extracts that were most effective were collected in areas of disturbed habitat adjacent to roads and are likely to have been recently introduced. In contrast, the extracts with weaker anti-EIAV activity were found in undisturbed, forested areas in North Carolina. Further genetic and metabolomic studies will be required to understand this potential constituent diversity within *Prunella vulgaris*.

## Abbreviations

EIAV: equine infectious anemia virus; ED: equine dermis cells; eUVEC: equine umbilical vein endothelial cells; HIV-1: human immunodeficiency virus-1; HSV-1: herpes simplex virus-1; ELR1: equine lentivirus receptor-1; DMSO: dimethyl sulfoxide.

## Competing interests

The authors declare that they have no competing interests.

## Authors' contributions

MAB was responsible for all of the EIAV studies that were performed. She organized and wrote the manuscript and generated the figures. MPW was responsible for the oversight of growth of the *Prunella *and participated in the harvesting and processing of the plant material. JAM was responsible for the collection and documentation of the *Prunella *accessions, the planting, maintenance of the plants, harvesting and processing of the *Prunella*. PM was responsible for the oversight of the *Prunella *fractionation and ethanol precipitations. CH was responsible for performing all fractionation of the *Prunella*. LR and BN were responsible for GC/MS and LC/MS analysis of the fractions and sub-fractions. WM was responsible for oversight of the project including design and coordination of the study. She was principally responsible for the editing of the manuscript.

## Supplementary Material

Additional file 1 **Supplementary figure 1**. Water extracts of *P. vulgaris *inhibit lentiviral infectivity with low cell toxicity. Click here for file
