# Supplementary material for: Inhibition of lentivirus replication by aqueous extracts of Prunella vulgaris
Source: Virol J. 2009 Jan 20;6:8. doi: 10.1186/1743-422X-6-8 (PMC2645367; doi:10.1186/1743-422X-6-8)
Supplement: Additional file 1 — Supplementary figure 1. Water extracts of P. vulgaris inhibit lentiviral infectivity with low cell toxicity. [file 1743-422X-6-8-S1.ppt]

## Slide 1
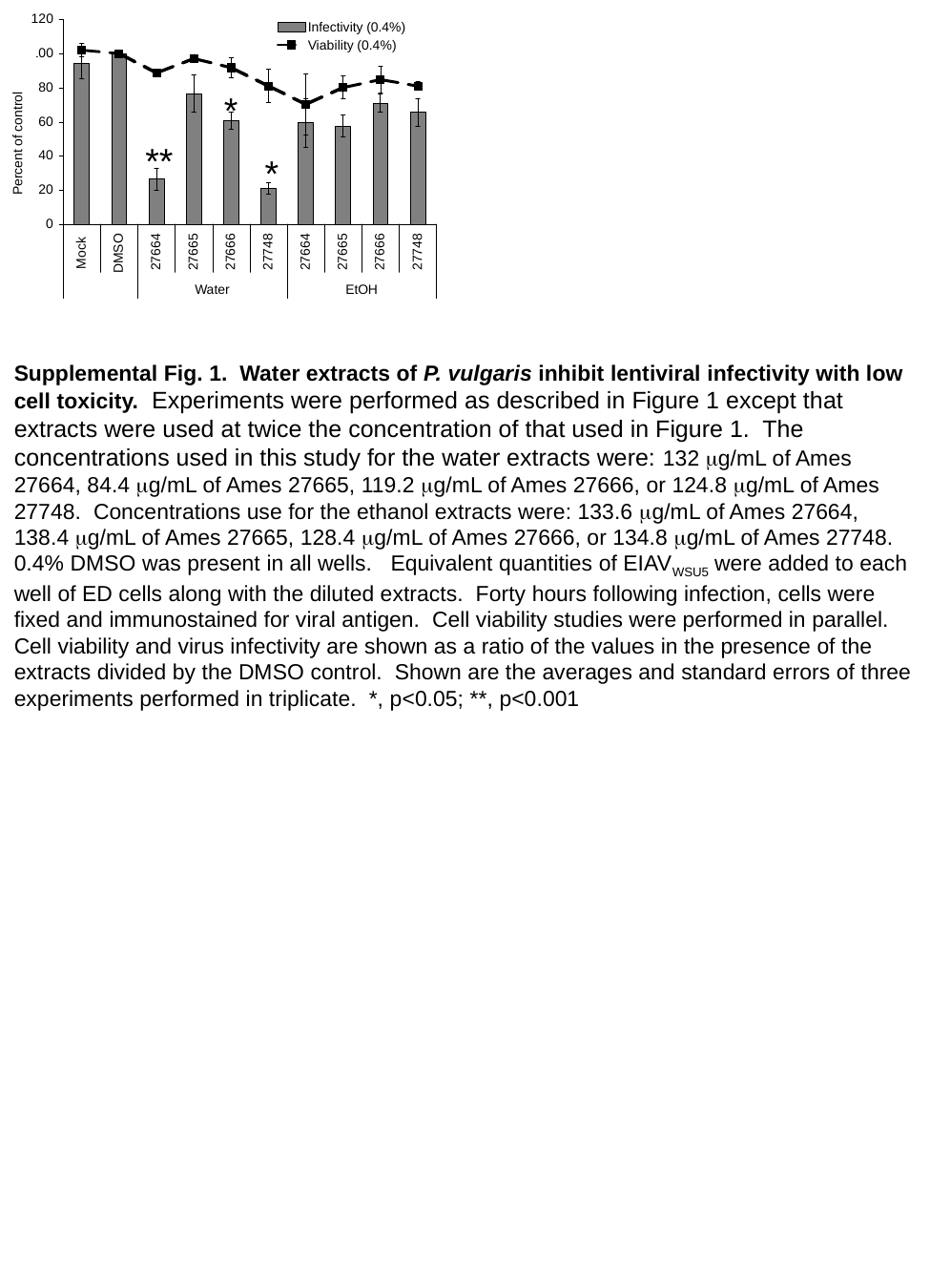

*
**
*
Supplemental Fig. 1. Water extracts of P. vulgaris inhibit lentiviral infectivity with low cell toxicity. Experiments were performed as described in Figure 1 except that extracts were used at twice the concentration of that used in Figure 1. The concentrations used in this study for the water extracts were: 132 μg/mL of Ames 27664, 84.4 μg/mL of Ames 27665, 119.2 μg/mL of Ames 27666, or 124.8 μg/mL of Ames 27748. Concentrations use for the ethanol extracts were: 133.6 μg/mL of Ames 27664, 138.4 μg/mL of Ames 27665, 128.4 μg/mL of Ames 27666, or 134.8 μg/mL of Ames 27748. 0.4% DMSO was present in all wells. Equivalent quantities of EIAVWSU5 were added to each well of ED cells along with the diluted extracts. Forty hours following infection, cells were fixed and immunostained for viral antigen. Cell viability studies were performed in parallel. Cell viability and virus infectivity are shown as a ratio of the values in the presence of the extracts divided by the DMSO control. Shown are the averages and standard errors of three experiments performed in triplicate. *, p<0.05; **, p<0.001
